# Absence of Subliminal Body‐Image Priming Effects in Anorexia Nervosa

**DOI:** 10.1002/eat.70148

**Published:** 2026-06-11

**Authors:** Jana Boettner, Benjamin Mosch, Sabine Steins‐Loeber, Martin Diers

**Affiliations:** ^1^ Clinical and Experimental Behavioral Medicine, Department of Psychosomatic Medicine and Psychotherapy LWL University Hospital, Ruhr University Bochum Bochum Germany; ^2^ Department of Clinical Psychology and Psychotherapy University of Bamberg Bamberg Germany

**Keywords:** anorexia nervosa, automatic evaluation, body representation, cognitive–affective processes, eating disorders, implicit processing, priming, subliminal

## Abstract

**Objective:**

Sociocultural and cognitive–neurobiological accounts suggest that automatic processing of body‐related cues may contribute to distorted body representation in anorexia nervosa (AN). This study tested whether subliminally presented body‐image cues modulate explicit evaluations of normal‐weight female bodies in women with AN compared to healthy controls (HC).

**Method:**

Seventy‐eight women (41 AN, 37 HC) completed a subliminal priming task in which normal‐weight female bodies were rated for attractiveness, desirability, and perceived normality following subliminal primes depicting bodies from different weight categories. Priming effects were indexed using bias scores (calculated as differences between ratings following normal‐weight versus other prime categories) and complemented by analyses of absolute target ratings. Data were analyzed using mixed‐design analyses of variance (ANOVAs) with Group and Prime Category as factors. Prime awareness was assessed using a detection task to ensure subliminal presentation.

**Results:**

Across all rating dimensions, the analyses revealed no significant main effect of Group and no Group × Prime Category interaction, indicating that subliminal primes did not modulate evaluations of normal‐weight bodies. In contrast, absolute ratings revealed robust main effects of group, with participants with AN providing consistently more negative evaluations than HC across all prime conditions. However, effect sizes for priming effects were small.

**Discussion:**

The findings suggest that explicit body‐related evaluations in AN reflect globally shifted evaluative baselines that operate largely independently of brief subliminally presented cues. These results delineate boundary conditions for subliminal priming effects and highlight the stability of body‐related evaluative processes in AN.

## Key Points

- No evidence was found that subliminally presented body‐image cues altered evaluations of normal‐weight bodies in women with anorexia nervosa or healthy controls.
- Women with anorexia nervosa showed consistently more negative body evaluations across conditions, independent of priming.
- Findings suggest that body dissatisfaction in anorexia nervosa may reflect stable evaluative patterns rather than heightened sensitivity to subliminally presented cues.

## 1 Introduction

Distorted body representation is core feature of eating disorders and pronounced in anorexia nervosa (AN; American Psychiatric Association 2022). Sociocultural models propose that internalization of the thin ideal contributes to distorted body representation by shaping maladaptive appearance‐related attitudes and evaluations (Frederick et al. 2022; Stice 2002). Growing evidence suggests that distorted body representation involves interacting cognitive and affective processes across multiple processing levels, including both controlled (i.e., conscious, deliberate) and automatic mechanisms (Dakanalis et al. 2016).

Current treatments for AN primarily target controlled cognitive and behavioral processes, including conscious appraisal and behavioral change (e.g., Fairburn 2008; Fairburn et al. 2013; Schmidt et al. 2014; Zipfel et al. 2015). However, high relapse rates suggest that maintaining mechanisms remain insufficiently addressed (Khalsa et al. 2017; Walsh 2013).

Dual‐process and habit‐based models propose that disorder‐compatible evaluations and behaviors become automatic and stimulus‐driven through associative learning processes operating partly independently of controlled regulation (Gawronski and Bodenhausen 2014; Steinglass and Walsh 2016).

Such mechanisms are particularly relevant in AN, characterized by persistent disturbances in body representation and interactions between automatic and controlled processing systems (American Psychiatric Association 2022; Beckmann et al. 2021; Dakanalis et al. 2016; Riva 2012). Evaluative biases are defined here as systematic deviations in the appraisal of body‐related visual stimuli, which may arise from these automatic processes. Examining processing outside of conscious awareness provides a stringent test of whether automatic body‐related mechanisms contribute to persistent evaluative biases.

Evidence for automatic body‐related biases in AN includes neuroimaging findings of altered reward processing and behavioral evidence (e.g., Boehm et al. 2018; Cserjési et al. 2010; Fladung et al. 2010). However, experimental evidence for subliminal body image priming remains limited. Subliminal body image priming refers to the brief, nonconscious presentation of stimuli intended to activate associated representations without explicit awareness (Kouider and Dehaene 2007; Leins et al. 2021; Van den Bussche et al. 2009). In healthy women, Leins et al. (2021) found no group‐level effects of subliminally presented body images on attractiveness or desirability ratings of normal‐weight bodies, although thin‐ideal internalization was modestly associated with bias scores (*r* ≈ 0.35–0.42). Consciously presented primes altered evaluations. Whether such cues influence evaluative judgments in individuals with AN remains unclear, particularly as the magnitude of evaluative shifts was associated with thin‐ideal internalization in that study.

The present study extends this paradigm to women with AN and healthy controls (HC), who rated normal‐weight bodies on desirability, attractiveness, and perceived normality following subliminal exposure to bodies across five body mass index (BMI) categories. This study follows a hypothesis‐testing approach. Based on evidence for altered reward responses, preferential evaluation of underweight stimuli, and increased automatic attentional bias towards such cues in AN (Fladung et al. 2010; Horndasch et al. 2018), we hypothesized that ultra‐thin and thin primes would decrease positive evaluations of normal‐weight bodies, whereas overweight and obese primes would increase such evaluations, with stronger effects expected in the AN group.

## 2 Methods

### 2.1 Participants

#### 2.1.1 Power Analysis and Sample Size

An a priori power analysis was conducted using G*Power (v. 3.1.9.6; Faul et al. 2007) for mixed‐design ANOVAs. Based on a conservative small interaction effect (*f* = 0.15; Leins et al. 2021), the required sample size was *N* = 76 (*α* = 0.05 and 1−*β* = 0.80). To account for potential exclusions, 91 participants (42 AN, 49 HC) were recruited.

#### 2.1.2 Recruitment and Eligibility

Participants with AN were recruited from inpatient and day‐clinic treatment at the Department of Psychosomatic Medicine and Psychotherapy, LWL University Hospital, Ruhr University Bochum (RUB). HC participants were recruited via online platforms and the student population. HC participants received financial compensation for their participation. Participants with AN were assessed as part of their clinical treatment and did not receive additional compensation. Inclusion was restricted to participants assigned female at birth due to the higher prevalence of AN in women (Hudson et al. 2007; Van Eeden et al. 2021).

Participants with AN required a clinician‐assigned diagnosis of AN. HC participants were excluded for a history of psychiatric disorders and BMI outside 18.5–24.9 kg/m^2^. After excluding four participants due to elevated detection performance (3 HC, 1 AN) and nine HC participants due to screening criteria, the final sample comprised 78 individuals (41 AN, 37 HC).

#### 2.1.3 Sample Characteristics

Groups did not differ in age (AN: *M* = 25.00, SD = 9.09; HC: *M* = 22.16, SD = 1.89), *W* = 696.00, *p* = 0.949, *r* = −0.01. BMI was significantly lower in AN (*M* = 16.01, SD = 2.55) than in HC (*M* = 21.07, SD = 1.37), *t*(56.90) = −10.80, *p* < 0.001, *d* = −2.46.

Among participants with available diagnostic subtype information (*n* = 37), AN diagnoses included restrictive (ICD‐10 F50.00; *n* = 15), active (F50.01; *n* = 15), and atypical (F50.1; *n* = 7) forms according to ICD‐10.

Common comorbidities included depressive, anxiety, trauma‐related, and obsessive‐compulsive disorders.

Race and ethnicity were not assessed, consistent with common research practice in Germany.

### 2.2 Procedure

Testing consisted of a single session (~30 min). After standardized instructions, participants completed a subliminal priming task followed by a detection task. Tasks were implemented in E‐Prime (Version 2.0.10.356, Psychology Software Tools, Pittsburgh, PA). Afterward, participants completed questionnaires. Testing was conducted individually in a quiet laboratory room at the hospital using a 15.6‐in. display.

### 2.3 Priming Task

The priming task was adopted from Leins et al. (2021), in which participants evaluated normal‐weight target bodies on three dimensions: attractiveness, desirability, and perceived normality. Responses were collected using a seven‐point Likert scale.

Each trial consisted of a fixation cross, a prime (16.67 ms), a scrambled mask (100 ms; Suslow et al. 2013), and a normal‐weight target body displayed for 4000 ms, during which participants provided ratings (Blechert et al. 2011).

Prime stimuli consisted of five computer‐generated bodies representing ultra‐thin, thin, normal‐weight, overweight, and obese categories (Figure 1). Ten normal‐weight body images served as targets. Each target was presented five times with randomly assigned primes, resulting in 50 trials.

**FIGURE 1:**
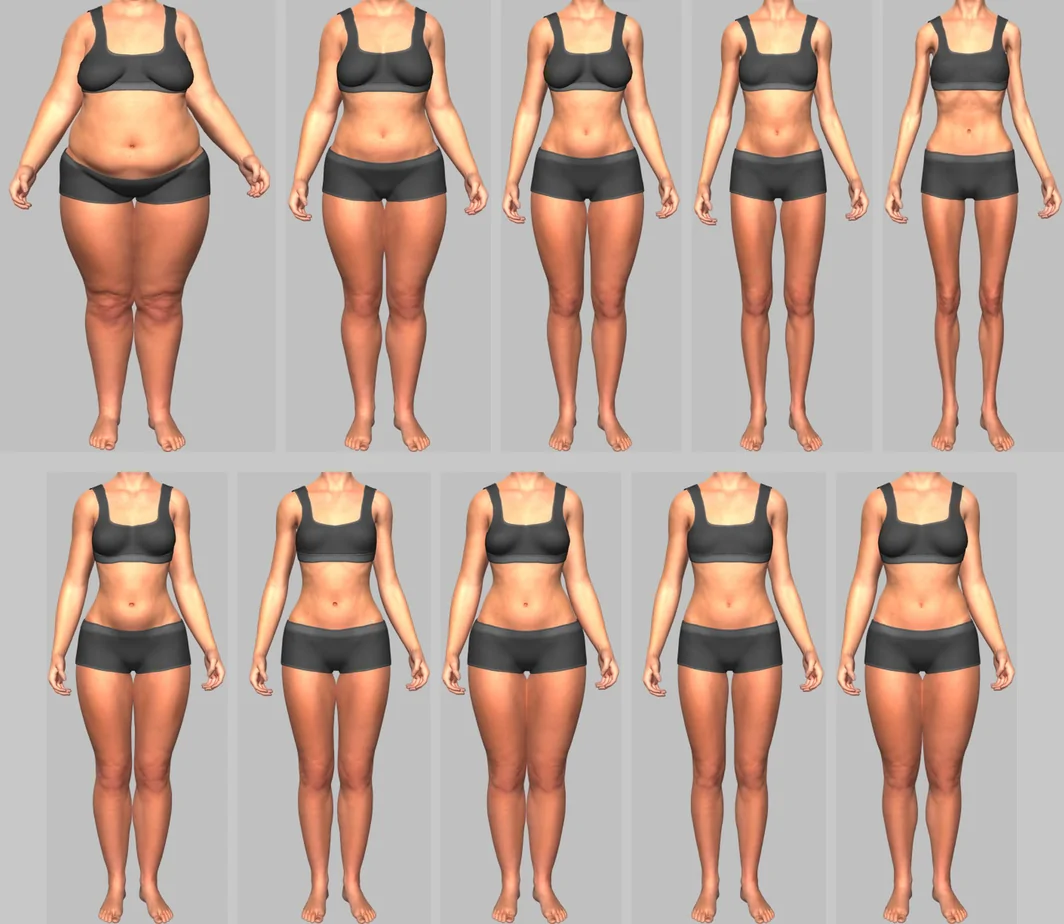
Computer‐generated female body stimuli used in the priming task. The upper panel displays the five body stimuli used as subliminal primes, representing distinct weight categories (from left to right: obese, overweight, normal weight, thin, ultra‐thin). The lower panel shows examples of normal‐weight target stimuli used in the priming task.

### 2.4 Detection Task

Participants were informed that primes had been presented subliminally and repeated the task, categorizing primes by weight using a five‐point scale (ultra‐thin to obese). Detection accuracy indexed prime awareness and served for outlier detection.

### 2.5 Questionnaires

Participants completed the Eating Disorder Examination Questionnaire (EDE‐Q; Fairburn and Beglin 1994), the Body Self‐Relation Questionnaire (FBeK; Strauß and Richter‐Appelt 1996), the Beck Depression Inventory (BDI; Beck et al. 1996), and the Sociocultural Attitudes Towards Appearance Questionnaire (SATAQ‐G; Heinberg et al. 1995; Knauss et al. 2009).

### 2.6 Statistical Analysis

Group comparisons of clinical and psychometric variables used Welch's *t*‐tests with Cohen's *d* when normality was met (Shapiro–Wilk test) and Wilcoxon rank‐sum tests with rank‐biserial correlations (*r*rb) otherwise. *p* values were Holm‐adjusted for multiple comparisons.

Questionnaire data showed partial missingness (4%–9%) that did not differ between groups (all Fisher's exact tests n.s.). Analyses were conducted using available cases. Rating data were complete.

Detection outliers were excluded using interquartile range (±1.5 × IQR) and median absolute deviation–based z score criteria (|*z*| ≥ 3.5). Remaining detection accuracy was tested against chance level (20%) using one‐sample Wilcoxon signed‐rank test.

Bias scores served as the primary outcome and were calculated by subtracting ratings following normal‐weight primes from ratings following the other prime categories. Priming effects were analyzed using mixed‐design repeated‐measures ANOVAs with group (AN vs. HC) as a between‐subjects factor and prime category as a within‐subjects factor. Absolute ratings were analyzed using analogous ANOVAs across all five prime categories.

Sphericity was assessed using Mauchly's test, and Greenhouse–Geisser corrections were applied when violated. Effect sizes are reported as generalized eta squared (*η* ^2^).

All analyses were conducted in R 4.5.0 (R Core Team 2024). Language editing support was provided using ChatGPT (GPT‐5, OpenAI; https://chat.openai.com) to improve grammar, wording, and readability of the manuscript.

### 2.7 Ethics and Data Availability

The study was approved by the ethics committee of RUB (approval no. 17‐6316 BR) and conducted in accordance with the Declaration of Helsinki. Data are openly available at: https://osf.io/gdar3.

## 3 Results

### 3.1 Clinical and Psychometric Characteristics

Participants with AN showed significantly higher scores on the EDE‐Q, SATAQ‐G, FBeK, and BDI‐II than HC (see Table 1).

**TABLE 1:** Group characteristics in clinical and psychometric variables.

	HC *n*	HC *M* (SD)	AN *n*	AN *M* (SD)	*p*	Effect size (*d*/*r*rb)
Demographics						
Age (years)	37	22.16 (1.89)	38	25.00 (9.09)	0.949	−0.009
BMI	37	21.07 (1.37)	38	16.01 (2.55)	< 0.001	−2.46
EDE‐Q Global	37	0.73 (0.66)	38	4.15 (1.32)	< 0.001	0.952
SATAQ						
Pressure	37	2.86 (1.15)	36	3.86 (0.95)	< 0.001	0.507
Internalization	37	2.40 (0.90)	36	3.82 (0.92)	< 0.001	0.719
Awareness	37	3.15 (0.70)	36	4.30 (0.57)	< 0.001	0.800
FBeK						
Insecurity/Discomfort	37	0.37 (0.17)	34	0.67 (0.13)	< 0.001	0.812
Attractiveness/Self‐Confidence	37	0.74 (0.17)	34	0.24 (0.16)	< 0.001	−0.912
Body Emphasis/Sensitivity	37	0.58 (0.16)	34	0.67 (0.10)	0.013	0.660
BDI Total	37	4.57 (4.52)	34	32.53 (10.62)	< 0.001	0.996

*Note:* Sample sizes vary across variables due to missing data (HC *n* = 37; AN *n* = 34–38). Reported *p* values are Holm‐adjusted. Group differences in BMI and the FBeK Body Emphasis/Sensitivity subscale were analyzed using Welch's *t*‐tests with Cohen's d as effect size; all remaining group comparisons were conducted using Wilcoxon rank‐sum tests with rank‐biserial correlations (*r*rb). Higher scores on the EDE‐Q (0–6) indicate greater eating‐disorder symptom severity. Higher scores on the SATAQ‐G subscales (1–5) reflect stronger endorsement of sociocultural appearance ideals. FBeK subscales (0–1) reflect stronger construct endorsement at higher values. BDI‐II scores range from 0 to 63 (0–13 minimal, 14–19 mild, 20–28 moderate, 29–63 severe depression).

Abbreviations: AN, participants with anorexia nervosa; BDI, Beck Depression Inventory; EDE‐Q, Eating Disorder Examination Questionnaire; FBeK, Body Self‐Relation Questionnaire; HC, healthy controls; M, mean; SATAQ‐G, Sociocultural Attitudes Towards Appearance Questionnaire (German version); SD, standard deviation.

### 3.2 Bias Scores

For attractiveness bias scores, no significant main effects of group (*F*(1, 76) = 0.60, *p* = 0.439, generalized *η* ^2^ (*η* ^2^_*G*) = 0.004), prime category (*F*(3, 228) = 0.20, *p* = 0.894, *η* ^2^_*G* = 0.001), or their interaction were observed (*F*(3, 228) = 1.19, *p* = 0.315, *η* ^2^_*G* = 0.007).

For desirability bias scores, no significant effects were observed for group (*F*(1, 76) = 0.51, *p* = 0.478, *η* ^2^_*G* = 0.004), prime category (*F*(3, 228) = 0.07, *p* = 0.978, *η* ^2^_*G* < 0.001), or their interaction (*F*(3, 228) = 0.29, *p* = 0.830, *η* ^2^_*G* = 0.002).

For perceived normal‐weight bias scores, no significant effects were observed for group (*F*(1, 76) = 0.02, *p* = 0.882, *η* ^2^_*G* < 0.001), prime category (*F*(3, 228) = 0.10, *p* = 0.962, *η* ^2^_*G* < 0.001), or their interaction (*F*(3, 228) = 0.86, *p* = 0.462, *η* ^2^_*G* = 0.005).

Effect sizes were small. Descriptive statistics are shown in Table 2.

**TABLE 2:** Absolute ratings and bias scores by prime category, group, and scale.

Scale	Prime category	Absolute ratings AN *M* (SD)	Absolute ratings HC *M* (SD)	Bias scores AN *M* (SD)	Bias scores HC *M* (SD)
Attractiveness	Ultra‐thin	3.27 (1.19)	4.88 (0.87)	−0.04 (0.33)	−0.05 (0.36)
	Thin	3.28 (1.19)	4.89 (0.84)	−0.03 (0.25)	−0.03 (0.28)
	Normal weight	3.31 (1.18)	4.92 (0.86)	—	—
	Overweight	3.32 (1.20)	4.80 (0.87)	0.01 (0.31)	−0.13 (0.37)
	Obese	3.27 (1.18)	4.86 (0.89)	−0.04 (0.38)	−0.07 (0.35)
Desirability	Ultra‐thin	2.75 (1.07)	4.52 (0.97)	−0.02 (0.36)	−0.01 (0.43)
	Thin	2.79 (1.08)	4.51 (0.90)	0.02 (0.31)	−0.02 (0.27)
	Normal weight	2.77 (0.98)	4.54 (0.91)	—	—
	Overweight	2.80 (1.07)	4.50 (0.94)	0.03 (0.33)	−0.04 (0.46)
	Obese	2.81 (1.04)	4.51 (0.93)	0.04 (0.37)	−0.03 (0.35)
Perceived normality	Ultra‐thin	3.84 (1.56)	5.60 (1.15)	−0.03 (0.38)	0.03 (0.41)
	Thin	3.86 (1.58)	5.55 (1.13)	−0.02 (0.29)	−0.02 (0.25)
	Normal weight	3.88 (1.58)	5.57 (1.10)	—	—
	Overweight	3.86 (1.58)	5.54 (1.13)	−0.02 (0.29)	−0.03 (0.26)
	Obese	3.89 (1.55)	5.52 (1.17)	0.02 (0.34)	−0.05 (0.37)

*Note:* Analyses are based on the full cleaned sample (AN *n* = 41, HC *n* = 37). Absolute ratings were provided on 7‐point Likert‐type scales ranging from 1 (not at all) to 7 (very much). Bias scores represent deviations from ratings following normal‐weight primes: negative values indicate lower ratings relative to the normal‐weight reference, whereas positive values indicate higher ratings.

Abbreviations: AN, Participants with anorexia nervosa; HC, Healthy controls; *M*, Mean; SD, Standard deviation.

### 3.3 Absolute Ratings of Target Body Stimuli

Absolute ratings reflect evaluations of normal‐weight targets following different prime categories.

For attractiveness ratings, a significant main effect of group was observed (*F*(1, 76) = 46.26, *p* < 0.001, *η* ^2^_*G* = 0.37), with higher ratings in HC than in AN. No main effect of prime category (*F*(4, 304) = 0.70, *p* = 0.592, *η* ^2^_*G* < 0.001) or interaction (*F*(4, 304) = 1.07, *p* = 0.372, *η* ^2^_*G* < 0.001) was observed.

For desirability ratings, a significant group effect was found (*F*(1, 76) = 62.64, *p* < 0.001, *η* ^2^_*G* = 0.44), with higher ratings in HC than in AN. No effects of prime category (*F*(4, 304) = 0.06, *p* = 0.994, *η* ^2^_*G* < 0.001) or interaction (*F*(4, 304) = 0.34, *p* = 0.854, *η* ^2^_*G* < 0.001) were observed.

For perceived normal‐weight ratings, a significant main effect of group was observed (*F*(1, 76) = 29.90, *p* < 0.001, *η* ^2^_*G* = 0.28), with higher ratings in HC. Because sphericity was violated for prime category, Greenhouse–Geisser corrections were applied. No effects of prime category (*F*(4, 304) = 0.15, *p* = 0.944, *η* ^2^_*G* < 0.001) or interaction (*F*(4, 304) = 0.70, *p* = 0.566, *η* ^2^_*G* < 0.001) were observed (see Table 2 and Figure 2).

**FIGURE 2:**
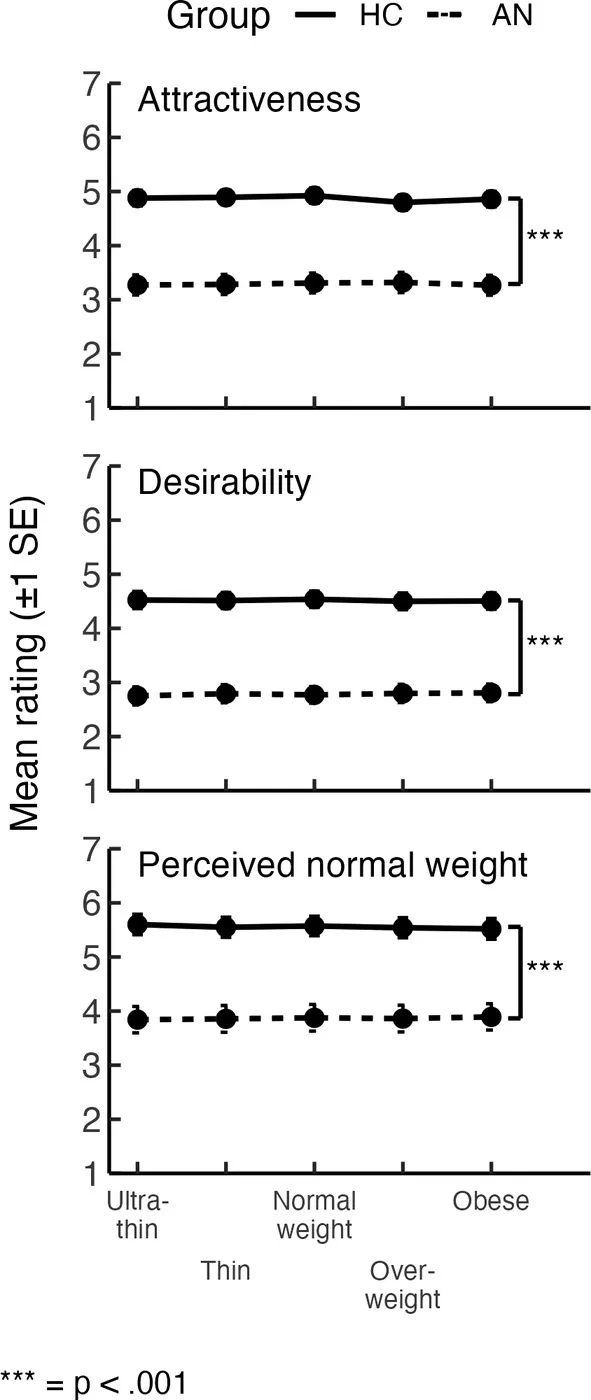
Absolute body image ratings across prime categories in HC and AN. Mean absolute ratings of attractiveness, desirability, and perceived normal weight for normal‐weight target stimuli following different prime categories (ultra‐thin, thin, normal weight, overweight, obese), shown separately for healthy controls (HC, solid line) and participants with anorexia nervosa (AN, dotted line). Error bars represent ±1 standard error of the mean. Brackets and asterisks indicate the significant main effect of group (*** *p* < 0.001).

### 3.4 Detection Task

After exclusions, detection accuracy was close to chance level (20%; *M* = 0.21, SD = 0.05), indicating no conscious prime categorization.

## 4 Discussion

### 4.1 Absence of Priming Effects

Contrary to our hypotheses, subliminally presented body images representing different weight categories did not significantly influence evaluations of normal‐weight female bodies in either group. No effects of prime category or group × prime interactions were observed across rating dimensions. Although participants with AN provided overall more negative evaluations than controls, these differences were independent of the priming condition, suggesting stable baseline differences rather than context‐dependent priming effects. This pattern is consistent with Leins et al. (2021), who likewise found no effects of subliminal body primes on explicit evaluations.

### 4.2 Limits of Subliminal Influences on Explicit Body Evaluations

The absence of subliminal priming suggests that implicit body‐related processes in AN may not readily translate into changes in explicit evaluations after brief nonconscious exposure. While previous research demonstrates altered reward responses to thinness during explicit stimulus processing (Fladung et al. 2010), and heightened affective reactivity to food cues following subliminal emotional priming (Soussignan et al. 2010), the present findings indicate that such processes may not necessarily influence explicit evaluations within the present paradigm. This interpretation aligns with cognitive–neurobiological models proposing that repeated reinforcement of thinness‐related goals produces durable associative links between thinness, control, and reward (Fladung et al. 2013; Steinglass and Walsh 2016).

### 4.3 Strengths, Limitations, and Future Directions

Methodological factors may also explain the absence of priming effects, although detection accuracy near chance supports effective masking. Subliminally presented body images may have lacked sufficient perceptual or emotional salience, and explicit rating measures may be less sensitive to subtle automatic influences than reaction‐time or physiological indices (Alexi et al. 2019; Cserjési et al. 2010; Moussally et al. 2017; Paslakis et al. 2021).

Strengths of the study include a well‐characterized clinical sample, rigorous control of prime awareness, and validated stimulus materials. However, inclusion of female participants only may limit generalizability.

Clinically, the findings suggest that subliminally presented body cues may have limited impact on explicit body evaluations in AN, underscoring the importance of interventions targeting consciously accessible cognitive and affective processes.

Future studies should combine behavioral, physiological, and neurobiological measures to better capture implicit mechanisms and clarify how automatic body‐related processes contribute to persistent body representation disturbances in AN.

## 5 Conclusion

The present study found no evidence that subliminally presented body images influenced explicit evaluations of normal‐weight bodies in women with AN or HC. Consistently more negative evaluations in AN suggest stable evaluative baselines and limited subliminal effects on explicit judgments.

## Author Contributions

**Martin Diers:** conceptualization, methodology, funding acquisition, formal analysis, project administration, resources, software, supervision, visualization, writing – original draft, writing – review and editing. **Sabine Steins‐Loeber:** conceptualization, methodology, software, writing – review and editing, validation. **Jana Boettner:** data curation, formal analysis, investigation, project administration, visualization, writing – original draft, writing – review and editing. **Benjamin Mosch:** investigation, project administration, writing – review and editing.

## Lived Experience Involvement Statement

No specific efforts were undertaken to involve persons with lived experience in the study design or execution, or in the preparation of this manuscript.

## Funding

This research was supported by Financial Resources of the Department.

## Conflicts of Interest

The authors declare no conflicts of interest.

## Data Availability

The data that support the findings of this study are openly available on the Open Science Framework (OSF) at https://osf.io/gdar3 (reference number gdar3).
